# Service user and carer involvement in mental health care safety: raising concerns and improving the safety of services

**DOI:** 10.1186/s12913-018-3455-5

**Published:** 2018-08-17

**Authors:** Kathryn Berzins, Gemma Louch, Mark Brown, Jane K. O’Hara, John Baker

**Affiliations:** 1 School of Healthcare, University of Leeds, Baines Wing, Woodhouse Lane, Leeds, LS29JT UK; 2 Yorkshire Quality and Safety Research Group, Yorkshire & Humber Patient Safety Translational Research Centre, Bradford Institute for Health Research, Temple Bank House, Bradford Royal Infirmary, Duckworth Lane, Bradford, BD9 6RJ UK; 3 Social Spider CIC, The Mill, 7-11 Coppermill Lane, Walthamstow, London, E17 7HA UK; 4 Leeds Institute of Medical Education, University of Leeds, Worsley Building, Leeds, LS2 9JT UK

**Keywords:** Health services research, Mental health services, Caregivers, Inpatients, Patient safety, Attitude of health personnel, Surveys and questionnaires

## Abstract

**Background:**

Previous research into improving patient safety has emphasised the importance of responding to and learning from concerns raised by service users and carers. Expertise gained by the experiences of service users and their carers has also been seen as a potential resource to improve patient safety. We know little about the ease of raising concerns within mental health services, and the potential benefits of involving service users and carers in safety interventions. This study aimed to explore service user and carer perceptions of raising safety concerns, and service user, carer and health professional views on the potential for service user and carer involvement in safety interventions.

**Methods:**

UK service users, carers and health professionals ( *n*= 185) were recruited via social media to a cross-sectional survey focussed on raising concerns about safety issues and views on potential service user and carer participation in safety interventions. Data were analysed using descriptive statistics, and free text responses were coded into categories.

**Results:**

The sample of 185 participants included 90 health professionals, 77 service users and 18 carers. Seventy seven percent of service users and carers reported finding it very difficult or difficult to raise concerns. Their most frequently cited barriers to raising concerns were: services did not listen; concerns about repercussions; and the process of raising concerns, especially while experiencing mental ill health. There was universal support from health professionals for service user and carer involvement in safety interventions and over half the service users and carers supported involvement, primarily due to their expertise from experience.

**Conclusions:**

Mental health service users and carers experience difficulties in raising safety concerns meaning that potentially useful information is being missed. All the health professionals and the majority of service users and carers saw potential for service users and carer involvement in interventions to improve safety, to ensure their experiences are taken into consideration. The results provide guidance for future research about the most effective ways of ensuring that concerns about safety can be both raised and responded to, and how service user and carer involvement in improving safety in mental health care can be further developed.

## Background

Improving patient safety has been a priority in health care for nearly two decades [1] and the subject of a significant body of research and innovation. In the UK, high profile cases of failures in health care, such as those in Mid Staffordshire NHS Trust have reinforced the need for this focus in general care. The resulting Francis Report [2] and the Keogh Report into quality of care across 14 other Trusts [3] particularly stressed the need for patient involvement in the monitoring and inspection of services and service responses to complaints. It has long been acknowledged that patient complaints are a crucial tool to facilitate service improvements [4]. The Parliamentary Ombudsman described a reluctance of people to make, and of Trusts to listen and respond to complaints, as a ‘toxic cocktail’ [5]. Figures from 2015 show that of all complaints made to Trusts very few are upheld (1.1% of all complaints to mental health Trusts and 1.8% of those to hospital Trusts).

Contrary to these developments in acute hospital and primary care settings, there has been little comparable research into the identification of safety issues in UK mental health care services despite serious failures in service provision [6]. This is particularly important given that it was a mental health Trust that was the first to be prosecuted post-Francis Report under new powers by the Care Quality Commission for failing to provide safe care and treatment [7]. One crucial aspect of improving patient safety is the potential for involving patients and their families as a resource for increasing safety by providing feedback either during or after their care experience. Patients and families are well positioned to notice things that staff do not [8] and provide an independent perspective based on their experiences of receiving care [9]. Evidence has shown that patients can willingly and meaningfully feed back on the safety of their care [10–14]. However, research on how to involve patients in interventions to improve safety and whether there are any benefits, is generally thought to be inconclusive [15]. Interventions have also involved patients directly intervening in their care, receiving education about their condition and feeding back about their experiences [16]. Research in acute hospital and primary care settings has found that patients are often willing to participate in interventions but are apprehensive about negative repercussions from raising concerns and question whether it is their responsibility to do so [17, 18]. One area that is relatively well developed in mental health care services is the principle of service user involvement which has been shown to improve several aspects of service, for example, better reflecting needs and preferences [19]. However, the context of mental health care is unique, service users can be at risk of compulsory treatment [20], experience high levels of stigma and low levels of social participation [21]. To date there has been minimal research carried out within a mental health care context around raising and responding to concerns and the involvement of service users and carers in safety interventions (Berzins K, Eames S, Newbronner L, Baker J, Thompson C: Service user involvement in interventions designed to improve safety in acute mental healthcare environments: a scoping literature review, submitted). Given the emergent evidence from acute hospital and primary care settings, service user and carer involvement could have an important role to play, but given the specific context of mental health care, evidence cannot simply be transferred. This study set out to explore how service users and carers felt about the ease of raising safety concerns, their views of involvement in safety interventions as well as those of health professionals, to understand how we might promote service user and carer involvement in safety to be taken forward by future research. This study had two aims:

1. To explore mental health service users’ and carers’ experiences of raising concerns about safety in mental health care services;
2. To explore service users’, carers’ and health professionals’ views on the potential for service user and carer involvement in future safety interventions.

## Methods

This paper reports data from a broader study that primarily sought to identify safety issues in mental health care, as such, more detailed information of the study method including that relating to procedure and participants has been reported previously [22].

### Design

A cross-sectional structured survey developed for this study was conducted with recruitment via social media (Twitter). This method of recruitment has been found to be effective in reaching potentially stigmatised groups [23]. Participants were eligible if they were over 18, and had recent experience (within the past 2 years) of using mental health care services, caring for someone using mental health care services, or experience of working in mental health care services.

### Data collection

Approval for the study was sought from the University of Leeds, School of Healthcare Research Ethics Committee (reference HREC15–059). At the beginning of the survey participants were given information about the study and sources of support. Consent was implied by completion and submission of the survey. The survey questions were informed by consultation with service users, carers and health professionals and the patient safety literature. The questions were focussed on the ease of raising concerns and reasons why; whether service user and carer participation should be part of future interventions to improve safety, reasons why or why not; and individual willingness to participate in such developments [24]. Questions took different formats, some used a Likert scale (In your experience how easy has it been to raise concerns about safety in mental health care? Very easy / easy / neither easy nor difficult / difficult / very difficult), some permitted multiple responses and others free text responses, which allowed respondents to provide examples and to elaborate on closed questions. To maximise completion of the survey, no responses were mandatory except two initial screening questions. Data were collected between September and December 2016.

### Data analysis

Quantitative data were analysed using SPSS [25]. Service users’ and carers’ views were analysed separately from health professionals’ views and descriptive statistics were performed to describe the characteristics of the two groups. For closed questions, results are presented in terms of percentages, and for questions that permitted multiple responses, results are presented in terms of percentage of cases. Data from questions that allowed free text responses were coded into categories, then counted to provide quantitative data and the original free text responses used to illustrate the quantitative findings.

### Findings

#### Sample characteristics

The demographic characteristics of the respondents are presented in Table 1. The sample of 185 consisted of 90 health professionals (49%), 77 service users (41%) and 18 carers (10%). There were more females than males in both groups (75%; *n* = 71 of service users and carers; 71%; *n* = 64 of health professionals) and the majority of participants described themselves as White British (76%; *n* = 72 of service users and carers; 72%; *n* = 65 of health professionals). Registered nurses were the largest health professional group (22%; *n* = 20) with managerial roles of different disciplines being the second largest (19%; *n* = 17).

**Table 1:** Sample characteristics

		Service users and carers % (n)	Health professionals % (n)
Age	18–25	15(14)	1(1)
	26–35	20(19)	18(16)
	36–45	19(18)	31(28)
	46–55	32(30)	37(33)
	56–65	13(12)	13(12)
	> 65	1(1)	0
Length of contact with services (years)	0–5	31(33)	14(16)
	6–10	26(27)	13(14)
	11–15	18(19)	17(19)
	16–20	4(4)	14(16)
	21–25	5(5)	9(10)
	26–30	6(6)	12(13)
	> 30	2(2)	11(12)
Type of service contact in past 2 years	CMHT^a^	45(47)	21(23)
	Inpatient services	16(17)	28(31)
	General Practitioner	24(25)	5(6)
	Voluntary organisation	6(6)	6(7)
	Other	3(3)	26(29)
Profession	Registered Mental Health Nurse	n/a	20(22)
	Managerial		17(19)
	Doctor		8(9)
	Psychologist		7(8)
	Lecturer		5(6)
	AMHP^b^		5(6)
	Researcher		5(6)
	Pharmacist		4(4)
	Care coordinator		3(3)
	Health Care Assistant		2(2)
	Support worker		2(2)
	Counsellor		2(2)
	Other		4(4)
	Missing		5(6)

^a^Community Mental Health Team

^b^Approved Mental Health Professional

Service users and carers were asked to describe their mental health problem or that of the person they cared for. Many participants gave multiple responses, most commonly, depression and anxiety (44%), post-traumatic stress disorder (17%) and personality disorder (16%). Forty three percent (*n* = 40) had experienced detention under the Mental Health Act.

The survey results are presented in two sections (1): service users’ and carers’ perceptions of ease of raising concerns; and (2) all participants’ perceptions of service user and carer involvement in interventions to address safety issues. Excerpts from the free text responses are used to further emphasise the key quantitative findings.

#### Ease of raising concerns

All participants were asked how easy they had found it to raise concerns about safety issues. A high rate of service user and carer participants (73 of 95; 77%) reported finding it very difficult or difficult to raise concerns (see Table 2).

**Table 2:** Participant responses regarding ease of raising concerns about safety

Group		Frequency	Percent
Service users and carers	Very easy	1	1.1
	Easy	4	4.2
	Neither	16	16.8
	Difficult	35	36.8
	Very difficult	38	40.0

The salient (in terms of frequency) service user and carer explanations for difficulty in raising concerns were that services do not listen (27% of cases; *n* = 22); concern about repercussions (19% of cases; *n* = 15); the process of raising concerns (16% of cases; *n* = 13); and the difficulty of raising a concern while experiencing mental ill health (14% of cases; *n* = 11). Several service users thought that their concerns were dismissed as being caused by their mental health difficulties. These difficulties were further supported by the information provided in the free text responses, as these excerpts illustrate:

‘If you raise any issues or challenge any decision you are seen as a difficult patient. You are not expected to have a valid viewpoint. They know best. They can make life very difficult, refuse to help you, and most likely change your diagnosis to personality disorder so that no one will want to treat you.’ (Service user #5).

‘I find it extremely hard to speak up for myself... When I have done I have been told because of my illness my opinions aren’t valid. I worry that my diagnoses render my opinions and suggestions worthless.’ (Service user #23).

‘It was difficult to raise concerns when I was on an inpatient ward; I was very distressed and very unwell so not easy to articulate concerns.’ (Service user #29).

#### Service user and carer involvement in interventions to address safety issues

The second aim of the study was to collect views about the potential for service user and carer involvement in interventions to increase safety in mental health care services, an area of rapid growth in other health care settings. There was a great deal of support for involving service users and carers in safety interventions by health professionals with 81% (*n* = 73) reporting that service users and carers should be involved, and the remaining 19% (*n* = 17) reporting that they should sometimes be involved. For both groups, the main perceived benefit of service user and carer involvement was that they can bring their expertise from experience to issues (Service users and carers 24%; *n* = 19 and health professionals 26%; *n* = 20).

‘I believe service users have a unique perspective that I don’t have.’ (Health professional #86).

Benefits perceived by health professionals were broader with the empowerment of service users (24%; *n* = 18) and the promotion of teamworking (18%; *n* = 14):

‘Because they are important allies in improving care.’ (Health professional #2).

When asked if they *personally* would be willing to be involved in safety initiatives, nearly all health professionals said they would be (91%; *n* = 82) with the remaining 9% (*n* = 8) reporting sometimes, depending on available support.

‘I would be keen to be involved but have so little time within work role I would not be able to commit to anything extra to role and would not receive managerial support to do so.’ (Health professional #134).

Health professionals’ reasons for personally participating echoed valuing the perspective of service users and carers (21%; *n* = 21), the potential for involvement to promote service user recovery (19%; *n* = 19) and team working (15%; *n* = 15).

‘I can’t think of a reason why I wouldn’t! Service users have a very important view of services, can see things a professional might miss/ignore.’ (Health professional #105).

Service users and carers did not feel quite as positive towards participation with 63% (*n* = 60) indicating that service users and carers should be involved, and just over a quarter saying they should sometimes be involved (27%; *n* = 26). Only 8% (*n* = 8) thought that service users and carers should never be involved. The reasons provided by those who suggested there should never be involvement of service users and carers included carers being too busy, and respondents not perceiving it as being the responsibility of service users and carers.

‘The onus should be on paid staff to take care of these things, without reliance on unwell people to plug gaps in the NHS.’ (Service user #121).

‘Carers have enough to do. This would relieve service providers and professional bodies of some of the responsibility.’ (Carer #8).

Participants who did think service users and carers should be involved in interventions to improve safety most frequently gave the same reason as health professionals, that their expertise could be informative (24%; *n* = 19). The next most frequent reason was that involving service users and carers might improve safety (23%; *n* = 18).

‘They should be involved if they want to be. Service users have a much greater insight into what is going on than staff often do.’ (Service user #100).

‘If patient safety is seen as the sole preserve of professionals they are partially sighted. They are missing a vital part of the picture.’ (Service user #122).

When asked whether they personally would be prepared to be involved in improving safety, service users and carers were less favourable than health professionals in their responses (all of whom indicated that they would always or sometimes personally support service user and carer involvement). Over half of the service users and carers (63%; *n* = 58) said ‘yes’, nearly a third (27%; *n* = 25) said ‘sometimes’ and 11% (*n* = 10) indicated ‘no’. Those who said they personally would be (sometimes) willing to participate suggested that was because their perspective was useful (27%; *n* = 19) and might contribute to improved safety (26%; *n* = 18):

‘Having felt unsafe so often I feel I have a lot to offer which staff could learn from.’ (Service user #100).

‘I would be happy to do anything to improve mental health services in general for future generations, myself and other service users. I think major change does need to happen.’ (Service user #5).

‘I am passionate about these issues as I am what I consider to be a “near miss”. I have been harmed by the system but remain committed to improving it for other service users, carers and for staff whom I have also seen harmed.’ (Service user #18).

Health professional participants agreed on the potential usefulness of service user and carer perspectives (26%; *n* = 20) followed by it being a potentially empowering experience for the service user or carer (23%; *n* = 18) and promoting collaborative working (18%; *n* = 14):

‘We need all the help we can get and it is important to enable patients and give them a voice but also respond when they raise issues. It should be a collaboration.’ (Health professional #31).

## Discussion

The aim of this study was to describe mental health care service users’ and carers’ views of raising concerns about safety in mental health care services, and to explore the potential for service user and carer involvement in future safety interventions. The findings show that service users and carers find it difficult to raise concerns about safety. The reasons service users and carers put forward to explain these difficulties were that services were perceived as neither listening nor responding; service users were concerned about repercussions; the very process of raising concerns was perceived as challenging; and the difficulty of raising a concern while experiencing mental ill health was considered problematic. There was universal support from health professionals and support from over half the service users and carers for service user and carer involvement in safety interventions, primarily due to their expertise from experience.

Inquiries into care failures in other health care settings have stressed the importance of listening and learning from issues raised by patients [2, 3], yet the findings of this study suggest we are some distance from achieving this in mental health care. Service users and carers described difficulties at all stages of the process, reluctance to initially raise a concern, concerns either not being listened to nor concerns being formalised but there being no response, but most striking was service users’ fear of repercussions. A major feature of the mental health care system is the presence of coercion, particularly in the hospital setting; a marked imbalance of power between health professionals and service users that might understandably make service users reluctant to voice concerns both at the time of the concern or retrospectively, as they might come back into contact with the same service on a regular basis. Some service users believed that their concerns would be dismissed as being caused by their illness, seen as delusional or even malicious, although research has shown this to be a rare occurrence [26, 27]. Many service users will have waited a long time to receive mental health care and may feel unable to raise concerns for fear of losing that service, meaning that services will not become aware of concerns and, therefore, be unable to respond. The fear of being seen as ‘difficult’, and the perceived threat of this contributing to a diagnosis of personality disorder, has no parallel in general health care; this was mentioned by more than one respondent and there is significant stigma attached, including inadequate treatment [28]. Furthermore, even if a service user or carer is prepared to raise an issue, it seems that the process may be difficult and not result in any change or even response, which may act as a barrier to future reporting and reduces the potential to learn from service user concerns about their experiences. These findings suggest that interventions aimed at improving safety by using concerns raised by service users and carers as a basis, may be more successful if service users and carers are directly involved in their development and implementation.

To date, there has been little empirical exploration of the willingness of service users and carers to participate in interventions designed to improve safety and also of the willingness of mental health professionals to collaborate to this end. The health professionals in this sample universally felt there was a role for service users and carers in such ventures and although there was not as high a level of support for involvement from service users and carers, nearly two thirds thought they should always be involved. Both groups thought service users and carers had expertise gained through their experiences and witnessing the experiences of others that they could contribute to interventions to improve safety. For example, service user perspectives on staff behaviour could inform interventions to reduce aggression; carers’ experiences of seeking information could change discharge procedures. This willingness to participate supports the findings in the general health care literature [12, 13], although there were similar reservations made about the main responsibility remaining with health professionals and there being occasions when service users would be too ill to participate [16].

A declared willingness to participate does not of course translate to meaningful involvement and those who participate may subsequently lack the diversity of the overall service user population [29]. This diversity within service users and carers means that there will need to be a diversity of methods used when striving for meaningful involvement in both research and subsequent safety improvement interventions [29] as well as offering opportunities for both short and long term involvement [30].

Given the paucity of existing evidence about the role of service users and carers as ‘co-producers’ of safety within mental health settings, one of the obvious implications of our findings is the need to co-design methods for the systematic and routine gathering of information about the safety of care and care services. However, those attempting to routinely gather feedback from service users and carers should proceed with caution. From the wider literature on the involvement of ‘patients’ in patient safety, it is evident that currently, there is no real consensus about the best way to achieve the systematic gathering of feedback about the safety of health care services, and that there may need to be multiple mechanisms if this is to be meaningful [30]. Indeed, some authors have recently described feedback from patients (service users) and carers as ‘soft intelligence’ that does not fit within existing mechanisms of gathering and acting on formalised metrics for managing services [31], or managing risk [29]. Further, current mechanisms for routinely collecting ‘patient feedback’ – such as the ‘friends and family test’ risk organisations believing that they are engaging with the views of those using services, when the reality is likely to be that they provide little or no information that can actually be used to improve services [32]. Thus, whilst our findings suggest that mental health care services would welcome feedback from service users and carers about safety to address concerns and improve services, operationalising this at a service or organisational level might not be straightforward. However, it is arguable that not collecting feedback represents a missed opportunity for organisational learning, as this type of information may well represent factors that may contribute to future patient safety problems [16], and as such, if collected routinely, may have the potential to inform services and improve the safety of mental health care.

### Strengths and limitations

This was a cross-sectional study using survey data. The sample contained more women and less than a quarter of participants came from minority ethnic groups. We already know that some ethnicities are over represented in mental health services, particularly in detentions under the Mental Health Act. It may be that these groups feel even more powerless to raise concerns. Therefore only limited generalisability of these findings can be claimed; however, in the absence of any knowledge about this area prior to this study, we do provide a starting point for more in-depth work in this area exploring the needs of particular groups such as people detained under the Mental Health Act and those with a range of other characteristics (gender; age; diagnosis, in particular complex trauma). Future research looking specifically at these groups is recommended as well as research looking at different service settings such as those for inpatients or community based services.

The sample was by its nature limited to those people using social media. There is also the tendency for people to link with organisations and individuals who reflect their own beliefs. Thus, this may mean the sample was biased further, although the on-line mental health community of interest has a broad scope. However, recruiting in this manner may also be a strength, as it allowed anonymous responses from service users and carers who might not have participated if recruitment had taken place directly through health services [22].

### Implications for future research

Future research might benefit from examining what the most effective ways are for ensuring concerns about safety from service users and carers can be both raised and responded to. For example, the Patient Measure of Safety (PMOS) [33, 11] is a theory based measure which captures the patient perspective of the safety of care. PMOS has been used in both general hospital care [11, 15], and has been adapted for use in a primary care setting [14]. One potential avenue for future research is to explore adapting PMOS for use in mental health care. An additional concern that would need to be explored is the risk that in empowering service users and carers to contribute to safety improvements and raising concerns, we may create inequalities associated with an effective exclusion of the most vulnerable groups. This is a concern that has been highlighted by authors examining patient involvement in acute care settings [29], but is likely to be even more of a concern and research priority in mental health care services. Finally, whilst initial research may focus on the systems required to support greater involvement from service users and carers, a wider question that will need to be explored in the fullness of time, is how service user and carer involvement in improving safety in mental health care can be developed at individual, service and system level.

## Conclusion

Overall, our findings suggest that mental health care service users and carers experience difficulties in raising concerns about the safety of health care services. Services were perceived to be unresponsive to complaints and service users feared repercussions if they raised a concern. This indicates that services are unlikely to receive this potentially useful data that could inform interventions to improve safety. All health professionals and the majority of service users and carers surveyed thought that there was potential for service user and carer involvement in developing interventions to improve safety informed by their experiences. The results of this study provide guidance for future research.

## Abbreviation

PMOS: Patient Measure of Safety
